# Visceral Obesity and Cytokeratin-18 Antigens as Early Biomarkers of Liver Damage

**DOI:** 10.3390/ijms241310885

**Published:** 2023-06-29

**Authors:** Giulia de Alteriis, Gabriella Pugliese, Antonella Di Sarno, Giovanna Muscogiuri, Luigi Barrea, Valentina Cossiga, Giuseppe Perruolo, Michele Francesco Di Tolla, Francesca Zumbolo, Pietro Formisano, Filomena Morisco, Silvia Savastano

**Affiliations:** 1 Endocrinology Unit, Department of Clinical Medicine and Surgery, University of Naples “Federico II”, Via Sergio Pansini 5, 80131 Naples, Italy; robiniapugliese@gmail.com (G.P.); antonella.disarno@gmail.com (A.D.S.); giovanna.muscogiuri@gmail.com (G.M.); francesca.zumbolo@gmail.com (F.Z.); sisavast@unina.it (S.S.); 2 Department of Humanities, Telematic University Pegaso, 80143 Naples, Italy; luigi.barrea@unina.it; 3 Diseases of the Liver and Biliary System Unit, Department of Clinical Medicine and Surgery, University of Naples “Federico II”, 80131 Naples, Italy; valentina.cossiga@gmail.com (V.C.); filomena.morisco@unina.it (F.M.); 4 Department of Translational Medical Science, University of Naples “Federico II”, 80131 Naples, Italy; gperruolo@gmail.com (G.P.); fpietro@unina.it (P.F.)

**Keywords:** visceral obesity, non-alcoholic fatty liver disease, Cytokeratin-18

## Abstract

Visceral obesity is linked to the progression of fatty liver to nonalcoholic steatohepatitis (NASH). Cytokeratin-18 (CK18) epitopes M30 (CK18M30) and M65 (CK18M65) represent accurate markers for detecting NASH. The aim of this study was to evaluate the association of CK18M30 and CK18M65 levels with anthropometric and metabolic characteristics, liver stiffness, and liver indices of steatosis and fibrosis in a cohort of subjects with visceral obesity; in this cross-sectional study, transient elastography (TE-Fibroscan^®^), anthropometric measurements, metabolic parameters, High Sensitivity C-Reactive Protein (hsCRP), and CK18M30 and CK18M65 levels (Apoptosense ELISA, PEVIVA, Germany) were evaluated. Fatty Liver Index (FLI), Fibrosis 4 (FIB-4), and Aspartate transaminase (AST)-platelet ratio index (APRI) were calculated; among 48 subjects, 47.2% presented metabolic syndrome, 93.8% hepatic steatosis, 60.4% high liver stiffness, and 14.6% hypertransminasemia, while FIB-4 and APRI were normal. CK18M30 and CK18M65 levels were significantly correlated with waist circumference, AST, ALT, HoMA-IR, liver stiffness, and APRI (*p* < 0.001). Subjects with CK18 fragments above the median values showed significantly higher waist circumference, HbA1c, AST, ALT, HoMA-IR, FLI, and APRI compared to those with values below the median; CK18M30 and CK18M65 levels correlated well with anthropometric and metabolic characteristics, representing good biomarkers for early identification of NASH in subjects with visceral obesity.

## 1. Introduction

Visceral obesity is an independent risk factor for non-alcoholic fatty liver disease (NAFLD), the hepatic manifestation of the metabolic syndrome (MetS) [1]. The deposition of triglycerides in the liver is a hallmark of NAFLD, which can progress, through an inflammatory phase defined as non-alcoholic steatohepatitis (NASH) towards fibrosis, cirrhosis, and hepatocellular carcinoma. In western countries, NAFLD became the principal cause of chronic liver disease due to the obesity epidemic [2,3]. The gold standard for diagnosing NASH is liver biopsy [4]. However, liver biopsy is an invasive procedure that is burdened with potentially significant complications. Thus, there is a great interest in noninvasive procedures to assist clinicians in diagnosing NASH and liver fibrosis. Imaging technologies, including Fibroscan and magnetic resonance elastography allow the assessment of fibrosis, but these procedures may be inaccurate in patients with obesity and should be integrated with other non-invasive tests.

The key event linked to the progression from steatosis to NASH with consequent fibrosis is the hepatocyte death by apoptosis [4]. Several blood-biomarkers have been studied for early identification of the progression to NASH. Cytokeratin-18 (CK18) is the main intermediate filament protein in hepatocytes that is released upon the initiation of cell death. In particular, the caspase-cleaved CK18 fragment M30 (CK18M30) is a marker of apoptosis, while M65, which includes both caspase-cleaved and intact CK18 (CK18M65), is a marker of necrosis [5]. Several studies support the use of circulating serum levels of CK18M30 and CK18M65 as accurate, noninvasive biomarkers to differentiate NAFLD from NASH [4,5,6]. However, despite the importance of obesity, particularly visceral obesity, and metabolic factors associated with the presence of advanced fibrosis and progression of liver injury in NASH, no studies have evaluated the association of circulating serum levels of CK18M30 and CK18M65 with anthropometric parameters, metabolic, and liver disease indices.

The aim of this study was to evaluate the association between serum levels of CK18M30 and CK18M65 with anthropometric measurements, biochemical parameters, liver stiffness values, and liver indices of steatosis and fibrosis in a cohort of subjects with visceral obesity to better characterize the metabolic profile of subjects with increased circulating serum CK18M30 and CK18M65.

## 2. Results

A total of 48 patients (42 females), aged 43.1 ± 14.2 yrs, met the inclusion/exclusion criteria and were included in the analyses. Table 1 reports all parameters analyzed in this study. Five of the subjects were overweight (9.4%), more than half (54.8%) had grade III obesity, and all had visceral obesity, but less than half (47.2%) met the criteria for the diagnosis of MetS according to NCEP ATP III criteria. Almost all the subjects (93.8%) had liver steatosis (58.3% severe steatosis), and 60.4% had a stiffness value higher than the normal values, but only 7 subjects (14.6%) had hypertransaminasemia. The FLI was higher than the normal values in all but one patient, and FIB-4 and APRI were in the normal range. Demographic, anthropometric, and metabolic parameters of the study population are reported in Table 1.

**Figure 1:**
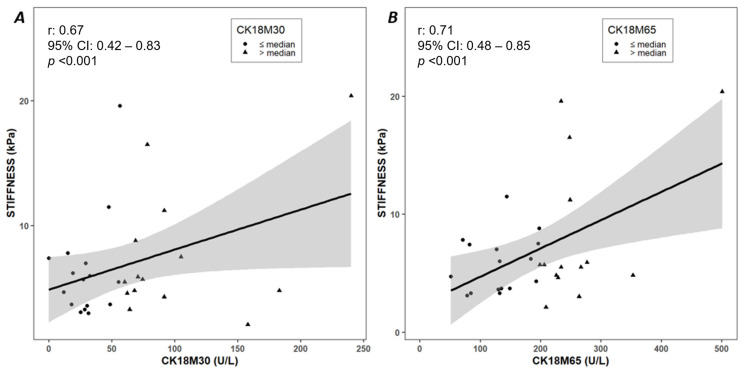
Correlations among CK18M30 (**A**) and CK18M65 (**B**) levels and liver stiffness. Pearson’s correlation was used to assess the association between the variables.

**Figure 2:**
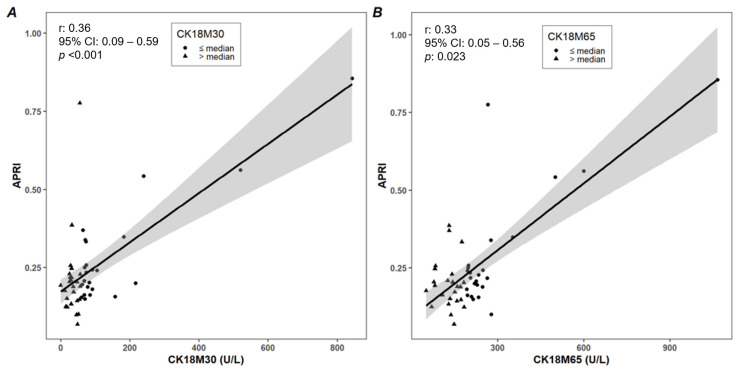
Correlations among CK18M30 (**A**) and CK18M65 (**B**) levels and stiffness and APRI (aspartate transaminase (AST)-platelet ratio index).

The significant bivariate correlations between the study variables and CK18M30 and CK18M65 levels are shown in Table 2; all the correlations are reported in Supplementary Tables S1 and S2. Both CK18M30 and CK18M65 levels exhibited significant correlations with anthropometric parameters, transaminases, liver stiffness, and HOMA-IR. In particular, the correlations among CK18M30 and CK18M65 levels, liver stiffness, and APRI are also reported in Figure 1A,B and Figure 2A,B, respectively.

Table 3a,b summarizes the differences in the study variables obtained by stratifying the population sample by the median values of CK18M30 and CK18M65. The subgroup with CK18M30 above the median value showed a higher prevalence of hypertension (*p* = 0.045), higher WC (*p* = 0.042), and ALT (*p* = 0.032) compared to the subgroup below the median value. The subgroup with CK18M65 above the median value showed higher AST (*p* = 0.018) and HoMA-IR (*p* = 0.036) compared to the subgroup below the median value. There was also a p-trend for WC (*p* = 0.042) and HoMA-IR (*p* = 0.059) for the subgroups with CK18M65 and CK18M30 above the median value, respectively. In both subgroups with CK18M30 and CK18M65 above the median values, HbA1c was higher than in the subgroup below the median value (*p* = 0.039 and *p* = 0.01, respectively), but no differences were evidenced in gender, prevalence of prediabetes, dyslipidemia, MetS, BMI classes, steatosis grade, and liver stiffness. In particular, differences in WC, HbA1c, HoMA-IR, FLI, and APRI between the subgroups with CK18M30 and CK18M65 above and below the median values are reported in Supplementary Figures S1–S3.

## 3. Discussion

The aim of this cross-sectional study was to detect the circulating levels of CK18M30 and CK18M65 fragments in a cohort of subjects with visceral obesity and to characterize their metabolic profile. The terminology and diagnostic criteria for NAFLD have recently been updated and a change in the nomenclature from NAFLD to MAFLD was proposed on the basis of the evidence that MetS is the most common and most serious etiology of NAFLD [2,7]. However, many studies and meta-analyses indicated that abdominal obesity measured by WC might lead per se to hepatic pathologic changes and represents a major risk factor for NAFLD [8]. Although NAFLD has been considered as the hepatic consequence of insulin resistance, the bi-directional relationships between visceral adiposity and NAFLD have shown that NAFLD can precede either type 2 diabetes and MetS, also representing a risk factor for their development [9]. Thus, the combined evaluation of serum biomarkers to differentiate NAFLD from NASH with anthropometric and metabolic parameters might be of key interest for phenotyping subjects with visceral obesity and NAFLD. In particular, CK18M30 detects apoptotic cell death, while CK18M65 detects both caspase-cleaved and uncleaved CK18, and thereby overall hepatocyte death [5]. As expected, hepatic steatosis was highly prevalent in our study population, affecting up to 93.8% of the subjects, 58.3% of which displayed a severe grade. In addition, 60.4% of the subjects presented increased liver stiffness evaluated by transient elastography. How ever, less than half of the subjects presented the criteria for MetS, and only 14.6% had hypertransaminasemia. Similarly, FLI, an algorithm representing a validated estimate of the pres ence of fatty liver and that incorporates both BMI and WC, was increased in all but one patient. However, FIB-4, a well-validated predictive score of liver fibrosis, and APRI, a non-invasive fibrosis scoring system, was in the normal range in all the subjects. As a novel finding, we combined the analysis of the anthropometric measures and the metabolic profile with the circulating levels of CK18M30 and CK18M65 fragments and the evaluation of liver steatosis and fibrosis. Our results showed direct associations between both circulating levels of CK18M30 and CK18M65 fragments and BMI, WC, insulin resistance evaluated by HoMA-IR, transaminases, liver stiffness, and APRI. In addition, subjects with higher values of circulating levels of CK18M30 and CK18M65 fragments presented higher HbA1c, FLI, and APRI, despite there being no differences in liver stiffness, likely due to the high prevalence of a degree of mild fibrosis (F1). Relative to visceral adiposity measured by WC and insulin resistance evaluated by HoMA-IR, there were some differences in the two subgroups, as the subgroup with higher CK18M30 showed higher WC and a trend of association with HoMA-IR, while the subgroup with higher CK18M65 showed higher HoMA-IR and a trend of association with WC. These associations let us hypothesize that high circulating levels of CK18M30 and CK18M65 fragments might allow early individuation of a subgroup of subjects with visceral obesity, more adverse metabolic profile, and increased risk of progression of NAFLD to fibrosis.

Early differentiation of NASH from NAFLD is of paramount relevance considering that NAFLD is becoming one of the leading indications for liver transplantation in western countries in the last 20 years [10]. However, while the diagnosis of NAFLD requires the presence of hepatic steatosis in radiological imaging and/or biopsy, after the exclusion of all other causes of chronic liver disease (e.g., alcohol consumption), the gold standard for the diagnosis of NASH still remains liver biopsy, which allows the identification of the pathognomonic features represented by ballooning, lobular inflammation, and steatosis [11]. However, liver biopsy is burdened by well-known and potentially life-threatening complications. A recently published systematic review and meta-analysis evaluating the diagnostic accuracy of blood biomarkers and non-invasive scores indicated that FLI and APRI were the risk scores with the highest prognostic value for the diagnosis of NAFLD and NASH, respectively [12]. Previously, the combination of CK18-Asp396 apoptotic fragment and Fibroscan improved their diagnostic performance for the detection of significant and advanced fibrosis in NAFLD [13] and a new scoring system for predicting NASH using the combination of this fragment with the FIB-4 index demonstrated a high predictive accuracy for the presence of liver fibrosis [14]. CK18M30 and CK18M65 fragments have been proven to significantly discriminate patients with fibrosis from healthy controls, although CK18M65 also provided a better diagnostic performance to differentiate low fibrosis stages [15]. The better performance for CK18M65 to detect NASH compared to CK18M30 was more recently confirmed by a systematic review and meta-analysis including 41 studies [6]. Of interest, in addition to transaminases, among the indices of liver steatosis and fibrosis included in our study population with visceral obesity, FIB4 and APRI were in the normal range, whereas FLI was higher than the normal values in practically all the patients. Adipocyte dysfunction is considered a key molecular link between visceral adipose tissue, MetS, and NAFLD [16], and larger deposits of visceral adipose tissue have proven to increase the 4-year risk of progression of incident NAFLD [17] and its progression to NASH [18]. Among the path ogenic mechanisms of NAFLD/NASH, visceral obesity might play a direct role in increasing the release of cytokines and other inflammatory mediators [19], or through the establishment of insulin resistance, might be an important factor in promoting the accrual of lipid droplets containing triglycerides, cholesterol esters, and other lipid species in the hepatocytes [20]. Although NAFLD pathogenesis has been extensively studied, the mechanisms associated with its progression to NASH are not fully characterized, but they include lipotoxicity and mitochondrial dysfunction promoted by lipid accumulation that triggers hepatocyte death, inflammation, and fibrosis [21]. Accordingly, in our study population, both the subgroups of patients with higher CK18 fragment levels also showed higher WC and APRI, with a significant difference in the subgroup with higher CK18M30 and a trend of association among those with higher CK18M65 fragment levels.

There are some limitations to this study that should be considered. The cross-sectional design of the study did not allow any causal association to be identified between visceral adiposity and circulating levels of CK18M30 and CK18M65 fragments and to clearly determine their prognostic value for early prediction of the risk of NAFLD progression to fibrosis. Furthermore, the sample size of patients with visceral obesity included in the study limited the extension of our findings to a population that shares different characteristics with our population sample. However, the sample size was calculated by using 95% statistical power that assured an adequate power to detect statistical significance on the results. In addition, although the single-center study design may have introduced some selection bias, the inclusion criteria allowed us to increase the homogeneity of the sample, as all the patients presented visceral obesity, were only naïve to treatment, and came from the same geographical area. We also recognize how liver biopsy is the gold-standard technique for identifying NASH. The disadvantage of the assessment of circulating levels of CK18M30 and CK18M65 fragments is that it is not routinely performed in daily practice, and the lack of a control group of subjects without visceral obesity represented further limitations to the study. In addition, only hsCRP was included among the inflammatory markers. However, FLI, liver stiffness, APRI, and CK18 fragments have largely been proven to represent easy and reliable screening tools to identify NAFLD/NASH in epidemiological studies. These possible drawbacks may prompt us to further investigate, in longitudinal studies, the association between visceral adiposity, circulating levels of CK18M30 and CK18M65 fragments, and inflammatory markers.

## 4. Materials and Methods

### 4.1. Design and Setting

This monocentric, cross-sectional study was carried out in patients with visceral obesity attending the Unit of Endocrinology, at the Department of Clinical Medicine and Surgery, University “Federico II” of Naples. The protocol was approved by the Federico II Ethical Committee (n. 468/20) and carried out in accordance with the Declaration of Helsinki (Code of Ethics of the World Medical Association) for experiments that involve humans. All the study participants signed an informed consent form after being clearly informed about the purpose of this research protocol.

### 4.2. Population Study

Consecutive adult subjects (aged ≥18 years) with visceral obesity and coming from the same geographical area around the Naples metropolitan area (Campania, Italy), were recruited for this study between January 2022 and January 2023. To increase the homogeneity of the patient sample, we included only treatment-naïve patients. Exclusion criteria were: patients with moderate to excessive use of alcohol, alcoholic liver disease, and liver diseases other than NAFLD; chronic kidney diseases; ischemic heart disease and heart failure; cerebrovascular diseases; asthma; rheumatoid arthritis; inflammatory bowel disease; and neoplasms.

### 4.3. Anthropometric Measurements and Blood Pressure

The patients’ weight was measured with a calibrated balance beam scale with the nearest 50 g (Seca 711; Seca, Hamburg, Germany). Their height was assessed using a wall-mounted stadiometer to the nearest 1 cm. Both for the measurements of weight and height, all participants wore light clothing and were without shoes when measuring. In accordance with the WHO’s criteria [22], body mass index (BMI) was calculated by weight (kg) and height squared (m^2^) and study participants were classified into three BMI classes: grade I obesity (BMI = 30.0–34.9 kg/m^2^), grade II obesity (BMI = 35.0–39.9 kg/m^2^), and grade III obesity (BMI =≥ 40.0 kg/m^2^; respectively). According to the NCHS, waist circumference (WC) was obtained using a non-stretchable measuring tape to the closest 0.1 cm at the narrowest point or to the nearest 0.1 cm at the umbilical level in patients with no narrowest point of the waist visible or with grade III obesity [23]. Blood pressure was measured 3 times in each arm using validated devices following the measurement protocol of the European Society of Hypertension and the European Society of Cardiology [24].

### 4.4. Assay Methods: Hormonal and Metabolic Profile

Blood samples were collected in the morning between 8 and 10 am after at least an 8-h overnight fast and stored at −80 °C until being processed. Fasting insulin levels were measured by a solid-phase chemi-luminescent enzyme immunoassay using commercially available kits (Immulite Diagnostic Products Co., Los Angeles, CA, USA). The intra-assay coefficient of variations (CV) fasting insulin levels (n. v. 1–20 μU/mL) was <7%. All biochemical analyses including fasting glucose, HbA1C, total cholesterol, triglycerides, alanine transaminase (ALT), aspartate aminotransferase (AST), and γ-glutamyltransferase (γGT) were performed with a Roche Modular Analytics System in the Central Biochemistry Laboratory of our Institution. HDL cholesterol and low-density lipoprotein (LDL) cholesterol were determined by a direct method (homogeneous enzymatic assay for the direct quantitative determination of HDL and LDL cholesterol). High sensitivity C-reactive protein (hsCRP) assay (cat. n. L2KCR2, Siemens, Washington, DC, USA) was performed using the IMMULITE^®^ 2000 Analyzer (DPC, Los Angeles, CA, USA), as previously described [25]. The intra- and inter-assay CVs were <7% for all biochemical assays performed. CK-18M30 and CK-18M65 serum levels were measured using the M30 and M65 Apoptosense ELISA, PEVIVA, Bünde, Germany). The intra-assay CVs of the CK18M30 and CK18M65 fragments were <10%. The lower limit of detection was 20 U/L and 11 U/L, respectively. According to the manufacturer’s instructions, the reference range provided reached a 95th percentile of 251 U/L and 431 U/L for CK-18M30 and CK-18M65, respectively.

### 4.5. Cardio-Metabolic and Liver Indices

MetS was diagnosed according to the NCEP ATP III definition [26]. According to Matthews et al. [27], the homeostatic model assessment-insulin resistance (HoMA-IR) was calculated and a value of >2.5 was used as the cut-off of insulin resistance. The fatty liver index (FLI) was calculated with the formula proposed by Bedogni G. et al. [FLI = eL/(1 + eL) × 100, L = 0.953 × loge triglycerides + 0.139 × BMI + 0.718 × logeγGT + 0.053 × WC−15.745]. An FLI value >60 indicated the presence of liver steatosis [28]. The fibrosis 4 (FIB-4) index to predict fibrosis was calculated as follows: (age (years) × AST (IU/L)/platelet count (109 /L) × square root of alanine transaminase (ALT) (IU/L)) [29]. The aspartate transaminase (AST)-platelet ratio index (APRI) was calculated using the following formula: ((AST(IU/L)/upper-limit of normal)/platelet count) × 100) [30].

### 4.6. Diagnosis of Liver Steatosis

The diagnosis of liver steatosis was defined by the ultrasound presence of increased echogenicity of liver parenchyma. The ultrasound examination was performed by two operators with expertise in abdominal/hepatic ultrasonography using a 3.5–5 MHz multifrequency probe (Logiq 7 Pro, GE Medical System). An NAFLD diagnosis was made in the presence of hepatic steatosis after the exclusion of other causes of chronic liver disease (alcohol con sumption, viral hepatitis, and autoimmune diseases). Steatosis was graded as follows: absent: normal liver echotexture; mild: slight and diffuse increase of liver echogenicity with normal visualization of the diaphragm and of the portal vein wall; moderate: moderate increase of liver echogenicity with slightly impaired appearance of the portal vein wall and the diaphragm; severe: marked increase of liver echogenicity with poor or no visualization of the portal vein wall, diaphragm, and posterior part of the right liver lobe [31].

### 4.7. Fibroscan and Controlled Attenuation Parameter Evaluation

Liver fibrosis was evaluated with non-invasive tests (NITs), comprising imaging techniques and biomarker scores. Therefore, the enrolled patients underwent transient elastography (TE) by Fibroscan^®^ and submitted a fasting blood sample in the same days [32]. Liver stiffness measurements (LSM) were performed by a single well-trained operator using a TE-Fibroscan instrument (502Touch, EchosenseTM, Paris, France). The results were expressed in kiloPascals (kPa) with a range from 2.5 to 75 kPa. The interquartile range (IQR) was defined as an index of the intrinsic variability of LSM. Only those measurements with more than ten successful acquisitions, a success rate of at least 60%, and an IQR range lower than 30% were classified as valid and taken into consideration for statistical evaluation. Stiffness threshold values were F1 ≤ 7.5 kPa; 7.5 < F2 ≤ 10 kPa; 10 < F3 ≤ 14 kPa; and F4 ≥ 14 kPa. According to controlled attenuation parameter (CAP) cut-offs expressed as decibels per meter, (dB/m) we classified the enrolled patients as S0, no steatosis (0–10% fat; 0–237 dB/m); S1, mild steatosis (11–33% fat; 238–259 dB/m); S2, moderate steatosis (34–66% fat; 260–292 dB/m); and S3, severe steatosis (>67% fat; ≥293 dB/m) [33].

### 4.8. Statistical Analysis

Data were assessed for normality with the D’Agostino–Pearson test. Skewed variables were normalized using logarithm transformation and re-converted into tables and figures. The mean ± standard deviation (SD), median (interquartile Range—IQR), or percentages (%) were used to present the data. Differences in study variables according to the median values of CK-18M30 and CK-18M65 were analyzed using Welch’s *t*-test or the Mann–Whitney test, when appropriate. The chi square (χ^2^) was used to evaluate the differences in the frequency distribution of categorical variables. Pearson’s correlation was used to assess the association between the variables. *p* values < 0.05 were considered to be statistically significant. Data were collected and analyzed using the RStudio version software 4.1.2 (https://www.R-project.org/, accessed on 1 November 2021) and software GraphPad 7.0 (GraphPad Software Inc., La Jolla, Ca). For the calculation of the sample size, we considered a 12.1% prevalence of NAFLD in the Campania Region. Considering a Type I/II error rate alpha 0.05, beta 0.1, and a power size of 95%, the number of subjects to be enrolled was found to be 41. Considering a drop-out rate of 15%, the minimum number of cases required was 47. The calculation of the sample size was performed using clinical software (https://clincalc.com/stats/samplesize.aspx, accessed on 1 November 2021).

## 5. Conclusions

In conclusion, combining the analysis of the anthropometric measures and the metabolic profile with the circulating levels of CK18M30 and CK18M65 fragments and the evaluation of liver indices of steatosis and fibrosis, we showed direct associations between both circulating levels of CK18M30 and CK18M65 fragments and visceral obesity, insulin resistance, transaminases, liver stiffness, and APRI. In addition, we found that subjects with the highest values of CK18M30 and CK18M65 fragments presented the highest values of visceral adiposity, insulin resistance, HbA1c, FLI, and APRI. These associations let us hypothesize that high circulating levels of CK18M30 and CK18M65 fragments might allow early individuation of a subgroup of subjects with visceral obesity, more adverse metabolic profile, and higher than normal indices of steatosis and fibrosis severity. Large-scale prospective studies are needed to establish whether this new information is sufficient to modify existing clinical practice.

## Figures and Tables

**Table 1:** Demographic, anthropometric, and metabolic parameters of the study population.

Parameters		Patients (N. 48)
**Age**		43.1 ± 14.2
**Sex**	Males (%)	6 (12.5%)
	Females (%)	42 (87.5%)
**Height (m)**		1.61 [1.57; 1.68]
**Weight (kg)**		103.5 [91.7; 116.3]
**BMI (kg/m^2^)**		40.21 [34; 43.1]
**BMI classes**	Overweight (%)	5 (10.4%)
	Class I Obesity (%)	9 (18.7%)
	Class II Obesity (%)	8 (16.7%)
	Class III Obesity (%)	26 (54.2%)
**WC Males (<102 cm)** **WC Females (<88 cm)**	121 ± 15.9 107.7 ± 14.3	
**Blood Pressure**		
**SBP (≤130 mmHg)**		127.5 [120; 140]
**DPB (≤85 mmHg)**		80 [75; 85]
**Fasting Glucose (n.v. ≤ 110 mg/dL)**		88 [80; 97]
**Fasting Insulin (n.v. 3–25 μU/mL)**		20.3 [11.2; 25.9]
**HbA1c** **(n.v. < 6.5%)**		5.6 [5.3; 5.8]
**HoMA-IR (n.v. < 2.5)**		4.34 [2.36; 5.8]
**Total Cholesterol (n.v. < 190 mg/dL)**		179 [156; 197]
**HDL-Cholesterol (males > 40 mg/dL; females >50 mg/dL)**		47 [40; 56]
**LDL-Cholesterol (≤130 mg/dL)**		108.3 [93.25; 125.65]
**Triglycerides (n.v. ≤ 150 mg/dL)**		112 [86; 166]
**AST (n.v. 0–34 U/L)**		19 [15; 25]
**ALT n.v. 0–55 U/L)**		21 [16; 32]
**γGT (n.v. 9–36 U/L)**		22 [13; 33]
**hsCRP (0.57–2.59 mg/L)**		0.57 [0.33; 0.91]
**Transient Elastography Parameters**		
**Liver Steatosis**	Absent (%)	3 (6.3%)
	Mild (%)	8 (16.7%)
	Moderate (%)	9 (18.7%)
	Severe (%)	28 (58.3%)
**Stiffness (n.v. 2–4 kPa)**		5.6 [4.35; 7.73]
**% patients ≥ 4 kPa**		60.4%
**% patients in different Stiffness categories**	F1 ≤ 7.5	38 (79.2%)
	7.5 < F2 ≤ 10	2 (4.2%)
	10 < F3 ≤ 14	4 (8.3%)
	F4 ≥ 14	4 (8.3%)
**CAP (n.v. < 237 dB/m)**		316 [273.25; 367]
**Steatosis and Fibrosis Liver** **I** **ndices**		
**FLI (n.v. < 60)**		93.6 [55.2; 99.04]
**n. patients > 60**		47
**FIB-4 (n.v. < 3.25)**		0.64 [0.17; 1.89]
**APRI (n.v. < 1)**		0.21 [0.16; 0.25]
**Cytokeratin-18**		
**CK18M30 (n.v. < 251 U/L)**		56.4 [31.5; 75.4]
**CK18M65 (n.v. < 413 U/L)**		188.5 [137.3; 230.3]

In Table 1 are reported demographic, anthropometric, and metabolic parameters, transient elastography parameters, liver indices of steatosis and fibrosis, and CK18M30 and CK18M65 levels of the study population. Results are expressed as mean values ± standard deviation (DS), or as median values (interquartile range), or percentages (%). Abbreviations: BMI, body mass index; WC, waist circumference; SBP, systolic blood pressure; PAD, diastolic blood pressure; HoMA-IR, homeostatic model assessment for insulin resistance; HDL, high-density lipoprotein; LDL, low-density lipoprotein; ALT, alanine aminotransferase; AST, aspartate aminotransferase; γGT, gamma-glutamyl transferase; hsCRP, high sensitivity C-reactive protein; CAP, controlled attenuation parameter; FLI, fatty liver index; FIB-4, fibrosis-4; APRI, aspartate transaminase (AST)-platelet ratio index; cytocheratin 18 Fragments, CK18M30 and CK18M65.

**Table 2:** Significant correlations between CK18M30 and CK18M65 levels with study variables.

Parameters	CK18M30		CK18M65	
	R	*p* values	r	*p* values
**BMI**	r = 0.66	***p* < 0.001**	r = 0.65	***p* < 0.001**
**WC**	r = 0.77	***p* < 0.001**	r = 0.76	***p* < 0.001**
**AST**	r = 0.95	***p* < 0.001**	r = 0.95	***p* < 0.001**
**ALT**	r = 0.98	***p* < 0.001**	r = 0.97	***p* < 0.001**
**Stiffness**	r = 0.67	***p* < 0.001**	r = 0.71	***p* < 0.001**
**HOMA-IR**	r = 0.92	***p* < 0.001**	r = 0.91	***p* < 0.001**
**APRI**	r = 0.69	***p* < 0.001**	r = 0.72	***p* < 0.001**

CK18M30 and CK18M65 levels showed significant correlations with anthropometric parameters, transaminases, stiffness evaluated by transient elastography, and HoMA-IR. Pearson’s correlation was used to assess the association between the variables. Abbreviations: Cytocheratin 18 fragments, CK18M30 and CK18M65. BMI, body mass index; WC, waist circumference; ALT, alanine aminotransferase; AST, aspartate aminotransferase; γGT, gamma-glutamyl transferase; CAP, controlled attenuation parameter; HoMA-IR, homeostatic model assessment for insulin resistance; FLI, fatty liver index; FIB-4, fibrosis-4; APRI, aspartate transaminase (AST)-platelet ratio index.

**Table 3:** (**a,b**) Differences in study parameters obtained by stratifying the population sample by the median values of CK18M30 and CK18M6.

**a**							
		**CK18M30 ≤ µe** **(n = 25)**	**CK18M30 > µe** **(n = 23)**	***p*** **-Values**	**CK18M65 ≤ µe** **(n = 24)**	**CK18M65 > µe** **(n = 24)**	***p*** **-Values**
**Age**		41 ± 13.4	45.7 ± 14.6	0.252 a	42.5 ± 13.7	44 ± 14.6	0.730 a
**Sex**	M (%) F (%)	1 (2.08%) 24 (50%)	5 (10.42%) 18 (37.5%)	0.063 χ^2^	2 (4.2%) 22 (45.8%)	4 (8.3%) 20 (41.7%)	0.383 χ^2^
**Prediabetes**	No (%) Yes (%)	16 (33.3%) 9 (18.8%)	13 (27.1%) 10 (8.3%)	0.769 χ^2^	17 (35.4%) 7 (14.6%)	13 (27.1%) 11 (22.9%)	0.238 χ^2^
**Hypertension**	No (%) Yes (%)	17 (35.4%) 8 (16.7%)	9 (18.7%) 14 (29.2%)	**0.045 χ^2^**	13 (27.1%) 11 (22.9%)	13 (27.1%) 11 (22.9%)	0.999 χ^2^
**Dyslipidemia**	No (%) Yes (%)	10 (32.3%) 8 (25.8%)	7 (22.6%) 6 (19.3%)	0.925 χ^2^	10 (32.2%) 7 (22.6%)	7 (22.6%) 7 (22.6%)	0.725 χ^2^
**Metabolic** **Syndrome**	No (%) Yes (%)	13 (27%) 12 (25%)	11 (23%) 12 (25%)	0.773 χ^2^	14 (29%) 10 (21%)	10 (21%) 14 (29%)	0.248 χ^2^
**BMI (kg/m^2^)**		39 [31.6; 42.5]	41 [34.7; 45]	0.297 a	38.5 [31.6; 42.1]	41.1 [36.3; 46]	0.103 a
	Overweight (%) Class I (%) Class II (%) Class III (%)	2 (4.2%) 6 (12.5%) 6 (12.5%) 11 (22.9%)	3 (6.2%) 3 (6.2%) 2 (4.2%) 15 (31.3%)	0.471 χ^2^	2 (4.2%) 7 (14.6%) 4 (8.3%) 11 (22.9%)	3 (6.3%) 2 (4.2%) 4 (8.3%) 15 (31.2%)	0.341
**WC (cm)**		109 [99; 120]	120 [109; 136]	**0.042 b**	111 [98.5; 120]	118 [106; 136]	0.054 a
**SBP (mmHg)**		130 [120; 138]	125 [120; 140]	0.998 a	125 [120; 130]	130 [120; 140]	0.656 a
**DBP (mmHg)**		80 [75; 85]	80 [80; 90]	0.223 a	80 [75; 85]	80 [80; 90]	0.406 a
**Fasting Glucose (mg/dL)**		85 [76; 94.5]	91 [81; 100]	0.224 a	84 [76; 96]	91.5 [83.5; 97.8]	0.227 a
**Fasting Insulin (mg/dL)**		17.7 [10.2; 24.7]	22.5 [18.5; 27.9]	0.098 b	17.4 [10.4; 22.4]	23 [17.3; 29.7]	0.055 b
**HbA1c (%)**		5.35 [5.2; 5.7]	5.6 [5.48; 5.83]	**0.039 b**	5.3 [5.2; 5.7]	5.7 [5.6; 5.85]	**0.01 b**
**HoMA-IR (** **n.v. < 2.5** **)**		3.36 [1.97; 5.47]	5.31 [3.79; 6.04]	0.059 b	3.27 [2.12; 4.46]	5.32 [4.01; 6.92]	**0.03 b**
**Total Cholesterol (mg/dL)**		179 [157; 196]	182 [156; 206]	0.847 a	183 [160; 197]	176 [155; 198]	0.861 a
**HDL-Cholesterol (mg/dL)**		48 [40.5; 58]	44 [39; 53]	0.507 a	47.5 [41.3; 58.8]	43.5 [38.3; 50.8]	0.18 a
**LDL-Cholesterol (mg/dL)**		107 [96.1; 126]	110 [84.6; 126]	0.95 a	110 [99.4; 127]	108 [84.7; 124]	0.672 a
**Triglycerides (mg/dL)**		103 [79; 166]	124 [95; 180]	0.154 b	103 [78.5; 161]	124 [92; 172]	0.236 b
**AST (U/L)**		17 [14.5; 22]	20 [16; 28]	0.059 b	18.5 [12.5; 23.8]	25 [16.5; 48.8]	**0.018 b**
**ALT (U/L)**		19 [13; 24]	26 [18; 36]	**0.032 b**	19 [14; 27.8]	26 [13.8; 42.3]	0.16 b
γ**GT (U/L)**		19 [13; 30]	25 [14; 34]	0.288 b	1.6 [1.05; 2.4]	2.8 [2.08; 3.23]	**0.005 b**
**hsCRP (mg/l)**		0.58 [0.33; 1.4]	0.65 [0.33; 1.3]	0.999 b	0.59 [0.33; 0.9]	0.61 [0.35; 1.38]	0.772 b
**b**							
		**CK18M30 ≤ µe** **(n = 25)**	**CK18M30 > µe** **(n = 23)**	***p*** **-Values**	**CK18M65 ≤ µe** **(n = 24)**	**CK18M65 > µe** **(n = 24)**	***p*** **-Values**
***Transient Elastography Parameters***							
**Liver Steatosis**	Absent (%) Mild (%) Moderate(%) Severe (%)	0 (0%) 2 (5.6%) 4 (11.1%) 11(30.6%)	1 (2.8%) 3 (8.3%) 2 (5.6%) 13 (36%)	0.587 χ^2^	0 (0%) 2 (5.4%) 4 (10.8%) 11 (29.7%)	1 (2.7%) 3 (8.1%) 2 (5.4%) 14(37.9%)	0.573 χ^2^
**Stiffness (v.n. < 4 kPa)**		5.5 [3.65; 7.2]	5.7 [4.6; 11.2]	0.401 b	4.95 [3.53; 6.83]	5.7 [4.8; 12.6]	0.138 b
**CAP (v.n. < 237 dB/m)**		292 [242; 300]	341 [289; 383]	0.159 b	310 [255;359]	323 [273; 379]	0.587 b
***Steatosis and Fibrosis Liver Indices***							
**Fatty Liver Index (v.n. 0–100)**		88.6 [57.8; 96.3]	97 [85.8; 99.3]	**0.042** b	89.7 [55.2; 96.4]	96.2 [86.5; 99.4]	**0.036** b
**FIB-4 (n.v. < 3.25)**		0.60 [0.20–1.03]	0.69 [0.17–1.89]	0.207 b	0.59 [0.73–1.89]	0.73 [0.32–1.13]	0.102 b
**APRI (n.v. < 1)**		0.19 [0.14–0.23]	0.23 [0.18–0.34]	**0.021** b	0.19 [0.14–0.23]	0.23 [0.18–0.32]	**0.027** b

In Table 3a,b are reported the differences in study parameters obtained by stratifying the population sample by the median values (interquartile range) of CK18M30 and CK18M65. Differences were analyzed using Welch’s *t*-test or the Mann–Whitney test when appropriate. The chi square (χ2) was used to evaluate the differences in the frequency distribution of categorical variables. Abbreviations: Cytocheratin 18 fragments, CK18M30 and CK18M65; BMI, body mass index; WC, waist circumference; SBP, systolic blood pressure; PAD, diastolic blood pressure; HoMA-IR, homeostatic model assessment for insulin resistance; HDL, high-density lipoprotein; LDL, low-density lipoprotein; ALT, alanine aminotransferase; AST, aspartate aminotransferase; γGT, gamma-glutamyl transferase; hsCRP, high sensitivity C-reactive protein; CAP, controlled attenuation parameter; FLI, fatty liver index; FIB-4, fibrosis-4; APRI, aspartate transaminase (AST)-platelet ratio index. a: unpaired *t*-test; b: Mann-Whitney test; χ^2^: Fisher’s exact test.

## Data Availability

The datasets generated during and/or analyzed during the current study are available from the corresponding author on reasonable request.

## Supplementary Materials

The supporting information can be downloaded at: https://www.mdpi.com/article/10.3390/ijms241310885/s1.
