# Supplementary material for: Visceral Obesity and Cytokeratin-18 Antigens as Early Biomarkers of Liver Damage
Source: Int J Mol Sci. 2023 Jun 29;24(13):10885. doi: 10.3390/ijms241310885 (PMC10341576; doi:10.3390/ijms241310885)
Supplement: Supplementary file 1 [file ijms-24-10885-s001.zip › ijms-2454325-supplementary.pdf]

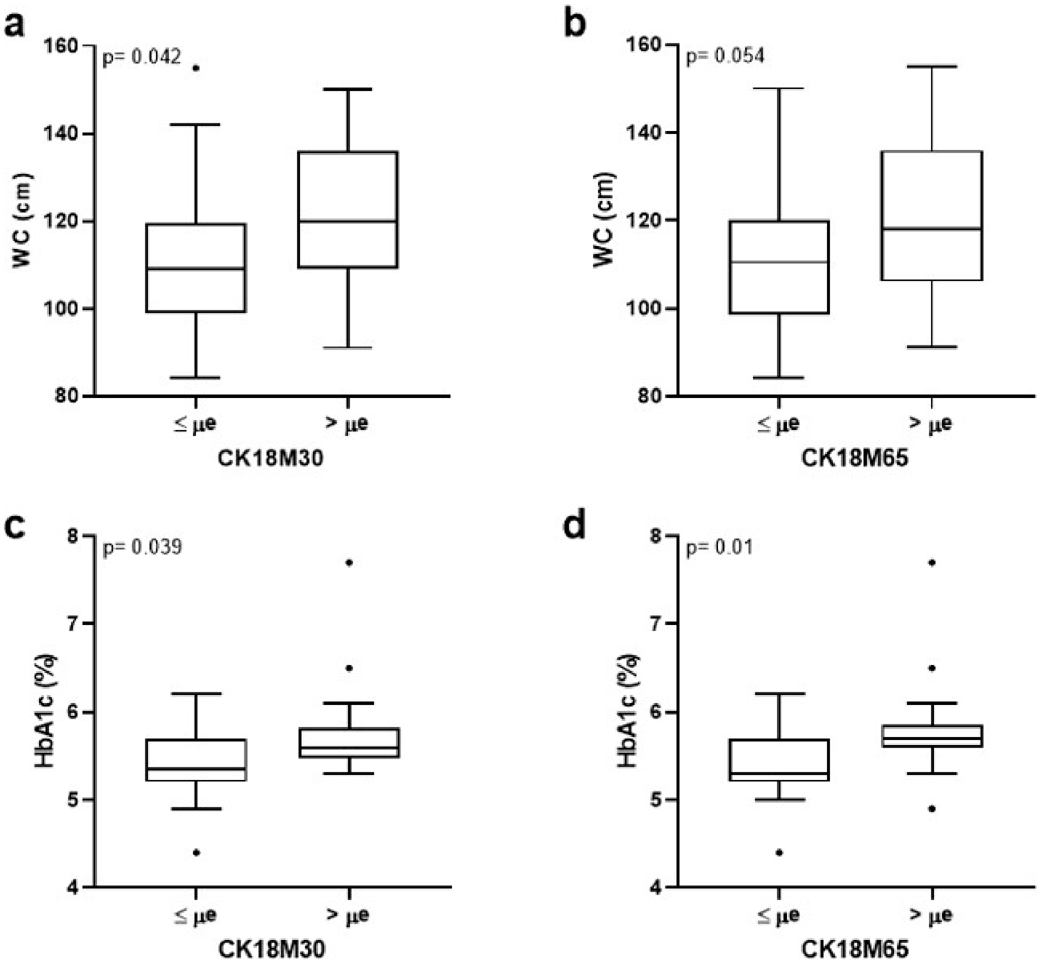

Supplementary Figure S1: Differences in WC (a and b, respectively), and HbA1c (c and d, respectively) between the subgroups with CK18M30 and CK18M65 above and below the median values. WC, Waist Circumference; Cytocheratin 18 Fragments, CK18M30 and CK18M65.

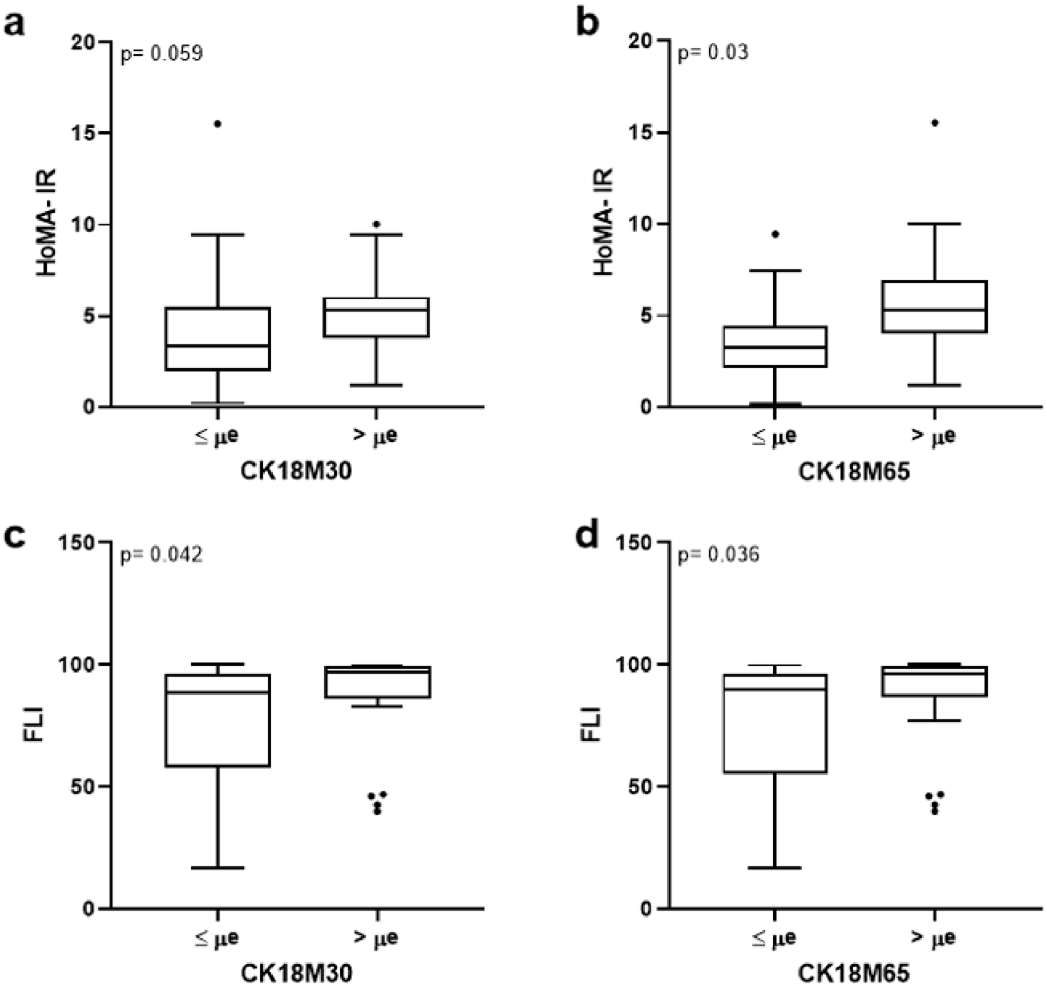

247

Supplementary Figure S2: Differences in HoMA-IR (a and b, respectively), and FLI (c and d, respectively) between the subgroups with CK18M30 and CK18M65 above and below the median values. HoMA-IR, Homeostatic Model Assessment for Insulin Resistance; FLI, fatty liver Index; Cytocheratin 18 Fragments, CK18M30 and CK18M65.

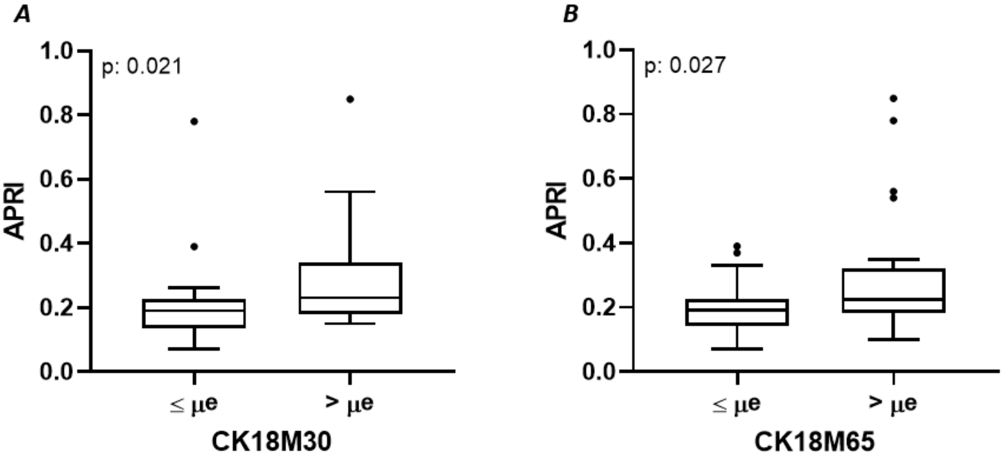

Supplementary Figure S3: Differences in APRI, between the subgroups with CK18M30 and CK18M65 above and below the median values (a and b). APRI, Aspartate transaminase (AST)-platelet ratio index; Cytocheratin 18 Fragments, CK18M30 and CK18M65.

Parameters	CK18M30		CK18M65	
	r	p-value	r	p-value
**Age**	r= -0.53	**p< 0.001**	r= -0.52	**p< 0.001**
**Height**	r= 0.65	**p< 0.001**	r= 0.64	**p< 0.001**
**Weight**	r= 0.97	**p< 0.001**	r= 0.96	**p< 0.001**
**BMI**	r= 0.66	**p< 0.001**	r= 0.65	**p< 0.001**
**WC**	r= 0.77	**p< 0.001**	r= 0.76	**p< 0.001**
**SBP**	r= -0.39	**p= 0.00381**	r= -0.4	**p= 0.00364**
**DBP**	r= 0.33	**p= 0.0374**	r= 0.36	**p= 0.03**
**Fasting Glucose**	r= 0.06	p= 0.145	r= 0.06	p= 0.155
**Fasting Insulin**	r= 0.96	**p< 0.001**	r= 0.95	**p< 0.001**
**HbA1c**	r= -0.05	p= 0.489	r= -0.04	p= 0.462
**HoMA-IR**	r=0.92	**p<0.001**	r=0.91	**p<0.001**
**Total Cholesterol**	r= -0.88	**p<0.001**	r= -0.89	**p<0.001**
**HDL-Cholesterol**	r= -0.92	**p<0.001**	r= -0.92	**p<0.001**
**LDL-Cholesterol**	r= -0.45	**p<0.001**	r= -0.44	**p<0.001**
**Triglycerides**	r= 0.78	**p<0.001**	r= 0.76	**p<0.001**
**AST**	r=0.95	**p<0.001**	r=0.95	**p<0.001**
**ALT**	r=0.98	**p<0.001**	r=0.97	**p<0.001**
**γGT**	r= -0.04	p= 0.851	r= -0.02	p= 0.782
**hsCRP**	r= 0.42	**p= 0.002**	r= 0.38	**p= 0.003**
**Stiffness**	r=0.67	**p<0.001**	r=0.71	**p<0.001**
**CAP**	r= 0.17	p= 0.36	r= 0.18	p= 0.32
**FLI**	r= 0.24	p= 0.27	r= 0.20	p= 0.28
**FIB-4**	r= 0.05	p= 0.72	r= 0.04	p= 0.79
**APRI**	r=0.69	**p<0.001**	r=0.72	**p<0.001**
**CK18M30**	-	-	r= 0.99	**p<0.001**
**CK18M65**	r= 0.99	**p<0.001**	-	-

Supplementary Table S1 : Correlations between CK18M30 and CK18M65 levels with evaluated parameters.
BMI, Body Mass Index; WC, Waist Circumference; SBP, Systolic Blood Pressure; PAD, Diastolic Blood Pressure; HoMA-IR, Homeostatic Model Assessment for Insulin Resistance; HDL, High-Density Lipoprotein; LDL, Low-Density Lipoprotein; ALT, Alanine aminotransferase; AST, Aspartate aminotransferase; γGT, Gamma-glutamyl transferase; hsCRP, high sensitive c-reactive protein; CAP, Controlled Attenuation Parameter; FLI, fatty liver Index; FIB-4, Fibrosis-4; APRI, Aspartate transaminase (AST)-platelet ratio index; Cytocheratin 18 Fragments, CK18M30 and CK18M65.

	**Stiffness**	**CAP**	**FLI**	**FIB-4**	**APRI**
**Stiffness**	1	r= 0.35<br>p= 0.054	**r= 0.37<br>p= 0.038**	r= 0.10<br>p= 0.607	**r= 0.5<br>p= 0.002**
**CAP**	r= 0.35<br>p= 0.054	1	**r= 0.38<br>p= 0.036**	r= 0.12<br>p= 0.518	r= 0.2<br>p= 0.240
**FLI**	**r= 0.37<br>p= 0.038**	**r= 0.38<br>p= 0.036**	1	r= 0.06<br>p= 0.768	r= 0.18<br>p= 0.220
**FIB-4**	r= 0.10<br>p= 0.607	r= 0.12<br>p= 0.518	r= 0.06<br>p= 0.768	1	**r= 0.35<br>p= 0.016**
**APRI**	**r= 0.5<br>p= 0.002**	r= 0.2<br>p= 0.240	r= 0.18<br>p= 0.220	**r= 0.35<br>p= 0.016**	1

Supplementary Table S2: Correlations between stiffness, CAP, FLI, FIB-4, and APRI
CAP, Controlled Attenuation Parameter; FLI, fatty liver Index; FIB-4, Fibrosis-4; APRI, Aspartate transaminase (AST)-platelet ratio index.
